# Preserved Thermal Pain in 3xTg-AD Mice With Increased Sensory-Discriminative Pain Sensitivity in Females but Affective-Emotional Dimension in Males as Early Sex-Specific AD-Phenotype Biomarkers

**DOI:** 10.3389/fnagi.2021.683412

**Published:** 2021-07-20

**Authors:** Toni Cañete, Lydia Giménez-Llort

**Affiliations:** ^1^Department of Psychiatry and Forensic Medicine, Autonomous University of Barcelona, Barcelona, Spain; ^2^Institut de Neurociències, Autonomous University of Barcelona, Barcelona, Spain

**Keywords:** pain, plantar test, biomarkers, nociception, sensory-discriminative, affective-emotional, translational research, gender medicine

## Abstract

The increase of the aging population, where quite chronic comorbid conditions are associated with pain, draws growing interest across its investigation and the underlying nociceptive mechanisms. Burn injuries associated problems might be of relevance in the older adult’s daily life, but in people with dementia, exposure to high temperatures and heat sources poses a significantly increased risk of burns. In this brief report, the hind paws and tail pain withdrawal reflexes and the emotional responses to thermal nociception in 3xTg-AD mice were characterized for the first time in the plantar test and compared to their non-transgenic (NTg) counterparts. We studied a cohort of male and female 3xTg-AD mice at asymptomatic (2 months), early (6 months), middle (9 months), and advanced (12 and 15 months) stages of the disease and as compared to sex- and age-matched NTg control mice with normal aging. At 20 and 40W intensities, the sensorial-discriminative thresholds eliciting the withdrawal responses were preserved from asymptomatic to advanced stages of the disease compared to NTg counterparts. Moreover, 3xTg-AD females consistently showed a greater sensory-discriminative sensitivity already at premorbid ages, whereas increased emotionality was shown in males. False-negative results were found in “blind to sex and age” analysis, warning about the need to study sexes independently. The current results and previous report in cold thermal stimulation provide two paradigms unveiling sex-specific early AD-phenotype nociceptive biomarkers to study the mechanistic underpinnings of sex-, age- and AD-disease-dependent thermal pain sensitivity.

## Introduction

Chronic pain is one of the most frequent causes of suffering and disability, affecting the individual’s quality of life. The increase of the aging population, where quite comorbid conditions are associated with chronic pain, draws growing interest across its investigation (Domenichiello and Ramsden, 2019). The neural processes of perceiving, encoding, and processing noxious stimuli (nociception) as well as the unpleasant sensory and emotional experience associated with actual or potential tissue damage (pain) define the two inseparable levels of study of one of the most relevant phenomena preserved until the very last seconds of life, becoming an important field of both neuroscience and medical research (Loeser and Treede, 2008). In addition, burn injuries’ sensitivity-related problems might be of relevance in the daily life of the older person. Older adults are one of the more susceptible populations to suffering burn injuries due to thinning skin and the aging process involving a progressive decline of sensory function (Jeschke and Peck, 2017). In people with dementia, exposure to high temperatures and heat sources poses a significantly increased risk of burns (Harvey et al., 2016). The neural processes of encoding and processing noxious stimuli differ from normal aging in these people. The sensory-discriminative dimension is maintained, increased pain tolerance is yet unclear, but the cognitive-evaluative and affective-emotional dimensions seem to be affected. During the progress of Alzheimer’s disease (AD) and other dementias, a difficulty of verbal expression ending in aphasia is added to the increasing severity of cognitive problems, with the consequent worsening or impossibility of trustful self-report. All these factors contribute to pain being under-detected and under-treated, a critical scenario still demanding important research efforts (Giménez-Llort et al., 2020).

Both in the clinical and basic research scenarios, the impact of pain in cognition has been largely studied, but how nociception and pain sensitivity are modified in individuals with several cognitive impairment levels is a research field that still needs more endeavors (Defrin et al., 2015). At the translational level, pain studies rely extensively on animal models to assess this process’s sensory and psychological complexities, and rodents are employed in the majority of this research (Giménez-Llort and Pick, 2017). Due to ethical considerations, research paradigms involving noxious stimuli elude those eliciting vocal expression of pain and relay in others based on withdrawal responses to avoid the stimulus, with a translation to those seen and assessed in humans through behavioral scales. For the investigation of thermal nociception in mice, the hot plate test’s withdrawal response requires both a supraspinal and a spinal mechanism. In contrast, a spinal reflex mediates the elicitation of the tail-flick and hind paws-withdrawal response following exposure to a heat stimulus in the radiant heat test (Plantar test or Hargreaves’s test), although supraspinal mechanisms can modulate this reflex (Chapman et al., 1985; Barrot, 2012). Pain discrimination measures in the radiant heat assay provide information about the pain experience’s sensory-discriminative aspect, yet they can yield some insights about the aversive or emotional aspect of pain (Mogil and Bailey, 2010).

The burden of pain is greater, more varied, and more variable for women than men (Cravello et al., 2019). Sex and gender differences in nociception and pain in aging are an important field of study. The understanding of these differences relays in biological (genetics and sex hormones), psychological (negative emotions, coping strategies), lifestyle, and socio-cultural factors that change across each individual’s life span (Berkley et al., 2006; Keogh, 2006). For at least the past decade, sex and age differences have been explored in some detail in rodent models (Giménez-Llort and Pick, 2017). However, this brief research report was aimed to characterize the sensory-discriminative and emotional responses to plantar and tail nociceptive thermal (heat) stimulation in male and female 3xTg-AD mice harboring PS1_M146V_, App_Swe_, tau_P301L_ transgenes (Oddo et al., 2003b) as compared to Non-transgenic (NTg) counterparts with the same genetic background (C57BL/6J×129/Sv). The novelty of studying pain responses in the 3xTg-AD model, at a spectrum of ages from young adulthood to old age, is that in the 3xTg-AD mice these ages mimic asymptomatic to advanced neuropathological stages of AD. In fact, this animal model of Alzheimer’s disease progressively develops βA plaques and neurofibrillary tangles with a temporal-and regional- specific profile that closely mimics their development in the human AD brain (Barrot, 2012). Synaptic dysfunction manifests in an age-dependent manner starting at 6 months of age but before plaque (12 months of age) and tangle (15 months of age) hallmark neuropathology (Belfiore et al., 2019) and severe cognitive impairment (Chapman et al., 1985; Giménez-Llort et al., 2007; Baeta-Corral et al., 2015). 3xTg-AD mice show diminished curiosity and apathy (exploration) in the open-field and lack of ability to cope with mild stressors (novelty) with increased emotionality (freezing behavior, defecation, and urination) in most of the behavioral tests (Oddo et al., 2003a, b; Billings et al., 2005; Giménez-Llort et al., 2007). At early and advanced stages of the disease, male 3xTg-AD show preserved tail flicking withdrawal response to a cold stimulus and presented a correlation with the immediate response exhibited in a sort of environments differing in the anxiogenic levels (Baeta-Corral et al., 2015).

## Materials and Methods

### Subjects

In a cohort of a total of 216 animals of our 3xTg-AD and NTg mice colonies established at the Behavioral Facility Core, Universitat Autònoma de Barcelona were used (Oddo et al., 2003b). Two to four littermates of the same genotype, age and sex were grown and maintained in macrolon cages (35 cm × 35 cm × 25 cm) under standard laboratory conditions of food and water *ad libitum*, in a temperature-controlled room 22 ± 2°C, a 12 h light/dark cycle (lights on at 8:00 a.m.) and relative humidity of 50–60%. A 2 × 2 × 5 experimental design included 55 male and 53 female 3xTg-AD mice at asymptomatic (2 months of age), early (6 months of age), middle (9 months of age), advanced βA (12 months of age) and tau (15 months of age) pathology stages of the disease that were evaluated and also compared to sex- and age-matched NTg control mice (52 males and 56 females). Animals were tested during the light phase, between 09:00 a.m. and 02:00 p.m., and following Spanish legislation on “Protection of Animals Used for Experimental and Other Scientific Purpose” and EU council directive (2010/63/UE) on this subject.

### Plantar Test—Hargreaves Test

Thermal sensitivity to noxious heat was tested using a Plantar test (Ugo Basile, Italy; Hargreaves et al., 1988) with a modification of the protocol to Cheah et al. (2017). Mice were habituated to the experimental room for at least 60 min before behavioral assessment. Following habituation, the animals were placed in a clear plastic chamber (a transparent Plexiglas box, 7.0 × 12.5 cm and 17.0 cm high) and left to acclimatize for 5 min before testing (habituate the animals to the testing chamber). During this time, the mice initially demonstrated exploratory behavior but subsequently stopped exploring and stood quietly with occasional grooming bouts.

**Figure 1:**
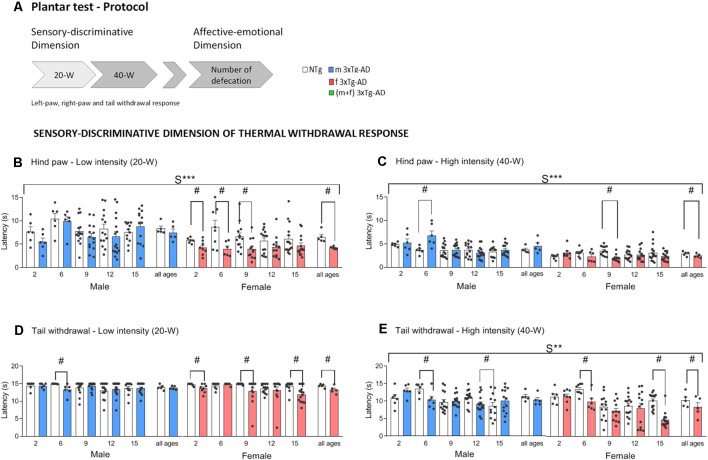
Schematic representation of the plantar test of thermal stimuli protocol used. Sensory-discriminative dimension of thermal withdrawal response in male and female 3xTg-AD and age-matched NTg mice with normal aging. **(A)** At low-20W and high-40W active intensity, infrared light stimuli were applied underneath the plantar surface of the mice’s hind paws (both right and left) and tail every 10 min. Paw (PWL) and tail (TWL) withdrawal latency were recorded for a sensory-discriminative dimension and the total number of defecations for an affective-emotional dimension. **(B)** Hind paw withdrawal responses at low-intensity 20-W **(C)** and high-intensity 40-W **(D)**. Tail withdrawal at low-intensity 20-W **(E)** and high-intensity 40-W. Groups: NTg, non-transgenic mice (open bar); 3xTg-AD, transgenic mice (male “black,” female “red” bar). Error bars represent mean ± SEM. Statistics: Sex effect, S***, *p* < 0.001 or S** *p* < 0.01. Student *t-*test comparisons are shown in the graphs as ^#^*p* < 0.05 vs. the NTg-group.

The sensitive-discriminative dimension of thermal pain was measured as follows: A mobile radiant heat source (infrared light stimulus), which was located 0.5 cm under the glass floor, was set to either 20% (Low-20W) or 40% (High-40W) active intensity halogen bulb. These heat stimuli were applied underneath the plantar surface of the mice’s hind paws (both right and left) and tail, through the base of the plastic box, every 10 min. Three alternate measures of withdrawal latency were established at 2 min-intervals in each paw, with a minimum of 2 s and a maximum of 15 s (cut-off latency). Paw and tail withdrawal latencies were recorded, and the three determinations were averaged for each animal. A cut-off latency of 15 s was imposed to avoid tissue damage. The affective-emotional dimension was measured as the total number of defecations, a classical measure of the animals’ emotionality, recorded until the end of the test (see Figure 1).

### Statistics

Statistical package for social science was used (SPSS, version 25.0 software). Data are expressed as mean ± SEM. Effects of factors genotype (G), sex (S), and age (A) in a 2 × 2 × 5 factorial design were analyzed with ANOVA followed by *Post hoc* Duncan’s multiple range test. In sex and age blind analysis, Student’s *t*-test was used. A probability value of less than 0.05 (*p* < 0.05) was considered statistically significant.

## Results

To avoid errors in the measurement and confounding factors that may elicit a higher sensitivity and response to thermal nociception (Barrot, 2012), it is important to bear in mind that different arousal and attention states, such as alert, resting, and light sleep, could be important modulators of pain sensitivity. Besides, it is important to avoid the testing while the subject is grooming (Callahan et al., 2008). Therefore, in this work, animals were acclimated to the testing apparatus. This procedure decreases the level of stress in the animal, its activity, and the variability in the data while not affecting the mean response times. As summarized in Figure 1A, measures were done in the left and right hind paws and tail of habituated animals using two intensities of the infrared light stimulus (20W and 40W).

**Figure 2:**
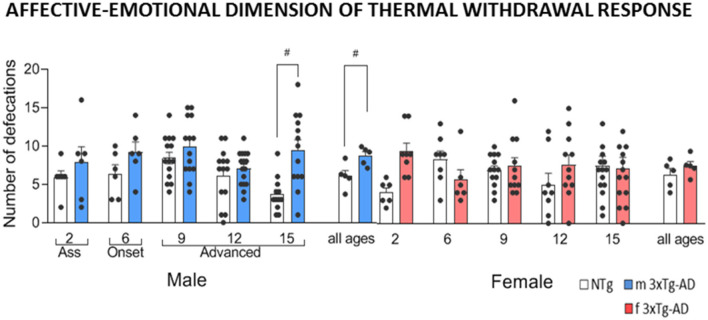
Affective-emotional dimension associated with thermal withdrawal response in male and female 3xTg-AD and sex- and age-matched NTg mice with normal aging. Total number of defecations. Groups: NTg, non-transgenic mice (open bar); 3xTg-AD, transgenic mice (male “black,” female “red” bar). Error bars represent mean ± SEM. Statistics: Student *t*-test comparisons are shown in the graphs as ^#^*p* < 0.01 vs. the NTg-group.

**Figure 3:**
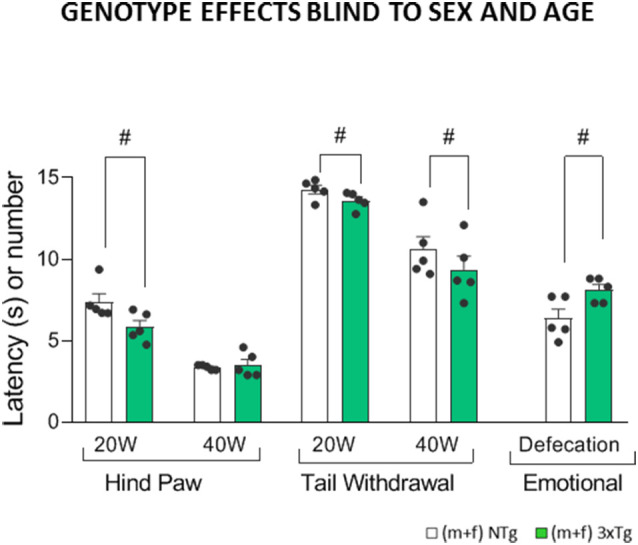
Genotype effects in the Sensory-discriminative and affective-emotional dimensions blind to sex and age in 3xTg-AD and NTg mice with normal aging. Represented by hind paw withdrawal responses at low (20-W) and high intensity (40-W), Tail withdrawal at low (20-W) and high intensity (40-W) and total defecations. Groups: NTg, non-transgenic mice (open bar); 3xTg-AD, transgenic mice (green bar). Error bars represent mean ± SEM. Statistics: Student *t*-test comparisons are shown in the graphs as ^#^*p* < 0.01 vs. the NTg-group.

Figures 1B–E and 2 depict the results per sex and age of the sensory-discriminative dimension of the thermal withdrawal response and the associated affective-emotional dimension, respectively. Figure 3 provides the global genotype analysis of these results in a sex and age blind analysis (pooled).

Age effects were found in hind paw withdrawal response latencies in both intensities (20-W; A, *F*_(4,215)_ = 2.49, *p* = 0.044 and 40-W; A, *F*_(4,215)_ = 2.56, *p* = 0.040), showing higher latency at 6 months of age compared to other ages. Tail withdrawal response also presented higher latencies at 2 and 6 months of age than at the rest of the ages (40-W; *F*_(4,215)_ = 8.98, *p* < 0.001).

Preserved thermal pain withdrawal responses in 3xTg-AD mice were found in both sexes during asymptomatic stages of the disease. Besides, in the case of females, 3xTg-AD mice exhibited shorter hind paw and tail withdrawal latencies than controls at the two intensities studied (Figures 1B–E, all ages, Paw-20W; #G (genotype effect), *t*_(107)_ = 4.30, *p* < 0.001. Paw-40W; #G, *t*_(107)_ = 2.42, *p* = 0.0017. Tail-20W; G, *t*_(107)_ = 3.39, *p* = 0.001. Tail-40W; G, *t*_(107)_ = 3.43, *p* = 0.001), indicating increased sensory-discriminative pain sensitivity.

Sex effects were found in hind paw withdrawal response latencies, in both low and high intensities (Figures 1B,C, 20-W; S***, *F*_(1,215)_ = 33.46, *p* < 0.001 and 40-W; S***, *F*_(1,215)_ = 31.63, *p* < 0.001) and the tail latency at 40-W (Figure 1E, S**, *F*_(1,215)_ = 10.09, *p* = 0.002). Further analysis per sex at each age indicated faster hind paw (20-W, #G, at 2, 6, and 9 months of age, *t*_(12)_ = 2.38, *p* = 0.035, *t*_(12)_ = 2.55, *p* = 0.025 and *t*_(23)_ = 2.55, *p* = 0.018 respectively; 40-W, #G, at 9 months of age, *t*_(23)_ = 4.42, *p* < 0.001) and tail (20-W, #G, at 2, 9, and 15 months of age, *t*_(12)_ = 2.12, *p* = 0.045, *t*_(23)_ = 2.15, *p* = 0.042 and *t*_(27)_ = 1.43, *p* = 0.001 respectively and 40-W, #G, at 6, and 15 months of age, *t*_(12)_ = 3.17, *p* = 0.008 and *t*_(27)_ = 0.17, *p* < 0.001) withdrawal responses of female 3xTg-AD mice as compared to controls. Also, in females, these genotype effects were already shown at asymptomatic ages (2 months of age) as unveiled by low intensity (hind paw, *t*_(12)_ = 2.38, *p* = 0.035 and tail *t*_(12)_ = 2.12, *p* = 0.045). At high intensity, shorter latencies were found at early (tail, 6 months of age, *t*_(12)_ = 3.17, *p* = 0.008) but also advanced (hind paw, 9 months of age, *t*_(23)_ = 4.42, *p* < 0.001; tail, 15 months of age, *t*_(27)_ = 0.17, *p* < 0.001) stages of the disease, respectively (Figures 1B–E).

Male 3xTg-AD only showed genotype effects in tail withdrawal response latencies, at early (20-W, #G, 6 months of age, *t*_(10)_ = 2.57, *p* = 0.028 and 40-W, #G, at 6 months of age *t*_(10)_ = 2.54, *p* = 0.029) and advanced (40-W, #G, at 12 months of age *t*_(28)_ = 2.33, *p* = 0.027) stages of the disease.

In summary, both low and high intensities, the withdrawal responses in hind paws (spinal reflex) and tail (spinal reflex subjected to supraspinal influences) consistently unveiled sex- and genotype-dependent effects. However, 3xTg-AD female mice consistently displayed shorter response latency to thermal stimulation than female NTg mice, already at the low-intensity, before advanced stages of disease (6- and 9-month-old) and at asymptomatic ages (2-month-old). Thus, the reduced thermal pain thresholds suggesting greater pain sensitivity were the hallmark of the withdrawal response of 3xTg-AD females as compared with their NTg counterparts.

Concerning the affective-emotional dimension, females and males exhibited increased defecatory behavior as compared to their respective age and sex-matched controls at early (female at 2 months of age, *t*_(12)_ = −3.85, *p* = 0.002) and advanced (male at 15 months of age, *t*_(23)_ = −3.82, *p* = 0.001) stages of the disease, respectively. However, the overall emotional response at all ages only reached statistical significance in males (*t*_(105)_ = −3.60, *p* < 0.001).

When results were studied blind to sex and age (Figure 3), false-negatives appeared since genotype effects were only detected in hind paw withdrawal response latencies at low intensity (20W; *F*_(1,215)_ = 10.23, *p* = 0.002). The tail’s withdrawal response was sensitive to genotypes at both intensities (20-W and 40-W; *F*_(1,215)_ = 5.93, *p* = 0.016 and *F*_(1,215)_ = 7.82, *p* = 0.006), always with a shorter latency in the 3xTg-AD animals as compared to controls. Regarding the emotional response, genotype differences were shown as an increased number of defecations in the 3xTg-AD mice (*F*_(1,215)_ = 10.42, *p* = 0.001) compared to controls.

## Discussion

Nociceptive pain is a function of the nervous system that provides important sensory information about our environment and external agents, being a protective mechanism for tissue damage. While special interest groups join efforts to improve pain assessment and intervention in older people with dementia is growing (Defrin et al., 2015; Domenichiello and Ramsden, 2019).

The main findings of the present study provided evidence that, under both 20W and 40W intensities studied, the sensory-discriminative threshold to a thermal stimulus in this cohort of male and female 3xTg-AD mice was preserved from the asymptomatic (2 months of age) to advanced stages of the disease (15 months of age) as compared to sex- and age-matched NTg mice with normal aging. Females exhibited increased sensory-discriminative pain sensitivity in both hind paw and tail, thus indicating spinal and supraspinal mechanisms’ involvement. In contrast, genotype differences in the emotional response were better shown in males. The results blind to sex and age elicited false negatives in the intensity of 40-W as the genotype effect was lost when pooling the sex and age groups.

The present results showing preservation of pain sensitivity to a hot stimulus through aging and AD processes complement those previously reported by our laboratory in the tail-flick test (Baeta-Corral et al., 2015). The nociceptive response to a cold stimulus was studied in male 3xTg-AD mice from early to advanced stages of disease (7 and 11 months of age, respectively) and found preserved. Tail-flick latency was only dependent on the age factor, and genotype effects were shown as a loss of functional correlation between the different behavioral components of the AD-phenotype. In the present work with the opposed thermal stimulus, we studied the plantar test response because it is a harmless and simple nociceptive reflex. This choice was determinant since in the hot-plate test female C57BL/6 mice show preserved thermal threshold at 3, 8, 15, and 28 months of age (Fahlström et al., 2011), but pain thresholds of female 3xTg-AD mice between 12 and 14 months of age did not differ from controls (Filali et al., 2012). Other studies indicate that wild-type mice’s pain sensitivity and emotional behavior increase with age, showing an important age-dependent effect on the hot-plate test. Instead, KO mice with a deletion of the p66 gene showed reduced pain sensitivity and emotionality (Berry et al., 2007). In the hot-plate, the TASTPM mouse model of AD exhibited reduced thermal sensitivity to acute noxious heat but also an increased opioidergic tone model (Aman et al., 2016). That study suggested increased inhibition/decreased excitation in the spinal cord as responsible for the reduced thermal sensitivity associated with AD-related pathology in this model. Similar studies in mutant mice with genetically-induced hypoglutamatergic tone, showed an hyposensitive phenotype to mechanical and thermal nociceptive stimuli, thus supporting the idea that glutamate plays a key role in physiological nociception both in acute and chronic pain (Kayser et al., 2015).

The present work supports the research about the basic sex differences in pain, with considerable implications for the clinic and how being blind to sex and age may lead to a false-negative. Some studies amply demonstrate the hypothesis on fundamental neural, hormonal, and developmental factors that mediate these sex/gender differences, which reinforces the conclusion that no single answer can explain sex/gender differences (Berkley et al., 2006). Currently, it is a clinical reality that women make up the large majority of chronic pain patients (perhaps 70%). The involvement of individual genes in pain-related traits—demonstrated *via* transgenic knockout mouse phenotypes or *via* quantitative trait locus (QTL; i.e., linkage) mapping—is sex-dependent on many occasions (Mogil and Bailey, 2010). Work in the laboratory animal examined screening for pain phenotypes and showed that C57BL/6 female mice were more sensitive than male mice, and similar sex differences were seen when analyzing the B6.MOLF12 congenic strain. The authors proposed that potential sex genotype interactions suggest sex-specific genetic linkages (Mogil et al., 2006). A review provides evidence of sex differences in pain sensitivity and the response to analgesic drugs in animals and humans. This summarizes that research on transgenic mice suggests that normal males have a higher level of activity in the endogenous analgesic system than normal females. It is postulated that males and females may have different pain modulatory circuits to suit these needs. Also, human study has found that μ-opioid receptors in the healthy female brain are activated differently from those in the healthy male brain (Kest et al., 2000). The response to k-opioids mediated by the melanocortin-1 receptor gene in both mice and humans is also different for each sex (Wiesenfeld-Hallin, 2005).

Pain discrimination measures provide information about the pain experience’s sensory-discriminative aspect but can yield little insight into the aversive aspect of pain or the affective-emotional dimension. The current finding of increased defecation in the 3xTg-AD mice genotype and mainly greater in 3xTg-AD males than the NTg group indicates a higher level of emotionality. In previous studies, increased emotional behavior was also described from early to advanced stages of disease when 3xTg-AD mice confronted anxiogenic conditions, from mild neophobia in the corner test or corridors resembling burrows in the T-maze to the classical open and illuminated field test, among others (Giménez-Llort et al., 2008, 2010, 2007). Despite being a controversial issue about rodent validity and reliability of defecation as an index of fear, there is sufficient evidence using a large sample of animals (Aguilar et al., 2003). In support of the pattern of sex effects reported here, about the male as being more fearful than females, on the one hand, confirms Gray’s view (Gray and Buffery, 1971), even a particularly relevant study with inbred Roman rats showed males defecated more than females in several different behavioral tests (Aguilar et al., 2003). On the other hand, it is known that a pattern of increased fecal bolis is found in stressed rats, along with increased plasma corticosterone concentrations (O’Mahony et al., 2009).

Anxiety and depression are related to pain, but their impact on emotionality may differ for males and females. In rats, it has also been observed that response to pain may determine other emotional behavior patterns, probably reflecting different activation thresholds of some brain structures controlling anxiety. They found that high-sensitivity male rats responded to an acute painful stimulation and showed significantly more freezing behavior. This suggests that animals that are more vulnerable to stress might have innate deficits in brain systems’ activity controlling the hypothalamic-pituitary-adrenal axis that would typically allow them to cope with stressful situations (Lehner et al., 2006). Female rats with maternal separation (a stressful process) had significantly longer paw lick latencies on a hot plate than handled rats, showing a persistent reduction in pain sensitivity. Therefore, the nociceptive circuitry involved in the hot plate’s responses appears particularly sensitive to early life stress (Weaver et al., 2007). Since our first reports (Giménez-Llort et al., 2006), 3xTg-AD mice show sex and age-dependent emotional and BPSD-like derangements, with neophobia fear-related decrease of rearing as the earliest behavioral marker emerging since early premorbid-like stages of disease (Giménez-Llort et al., 2007). For almost all behaviors assessed, male mice appear more fearful or deranged under fearful experimental conditions than females. The biological basis for these BPSD-like patterns was correlated to intraneuronal βA immunoreactivity in the basolateral amygdala (España et al., 2010).

Glucocorticoid levels have been inversely correlated with risk-taking behaviors and found strongly increased in young adult females compared to age-matched males. Although corticosterone was not measured in the present work, we have previously reported the sexual dimorphism in HPA axis activity in these NTg mice, as measured by higher plasmatic corticosterone levels in females than males at 6 months of age, and its strong reduction in old animals, due to an age-related decrease only observed in 15-month-old females but not male (Giménez-Llort et al., 2008). In contrast, like in the AD-human patients (Rasmuson et al., 2002), the corticosterone levels of 15-month-old 3xTg-AD mice were higher than in age-matched NTg mice, but sexual dimorphism was lost due to the increase of basal levels in males (Giménez-Llort et al., 2008). Furthermore, age-dependent sexual dimorphism in cognition and stress response in the 3xTg-AD mice has also been reported by other laboratories (Clinton et al., 2007). Therefore, the plasmatic corticosterone pattern would agree with the patterns of emotionality recorded here: increased emotionality in 2-month-old 3xTg-AD females but 15-month-old males.

Finally, the most recent report in humans shows that cognitively healthy apolipoprotein E4 (APOE4) carriers, with a demonstrated increased risk of late-onset AD, displayed lower overall pain sensitivity non-carriers but greater unpleasantness to thermal pain stimuli. This association of APOE4 allele status with an altered response to pain in a cognitively healthy sample of adults suggests that thermal evoked pain testing could serve as a potential phenotypic biomarker of individuals at increased risk for AD (Romano et al., 2021). The present work on the preserved withdrawal response in the 3xTg-AD mice since young age mimicks asymptomatic stages of disease agrees with this clinical finding and proposes sex-specific phenotypic biomarkers sensory-discriminative dimension in the female sex and affective-emotional in males.

Because the thermal pain measures were simply flat 20W and 40W intensity measures, the study has the limitation of not fully describing threshold intensity of limb/tail withdrawal. The study of only a mild thermal pain model may also seem one-dimensional to cover sensory-discriminative responses. Therefore, future studies may better elucidate incremental pain threshold response. On the other hand, in order to reduce to minimum confounding factors a cohort of animals of different ages, grown and aged under the same conditions, was assessed simultaneously. For this reason, despite the large total number of animals used, the number of each experimental group could not be larger. Whereas the results in females were very consistent, this limitation could be considered as compromising behavioral data in those results where intragroup variability would benefit of a bigger sample size.

In conclusion, the present work characterized, for the first time, sex- and age/AD-stage differences in the sensory-discriminative pain threshold to thermal pain stimuli measured as the withdrawal response of the left and right hind paws and tail in the plantar test in male and female 3xTg-AD mice and as compared to sex- and age-matched NTg counterparts with normal aging. We also provided evidence of easy-to-measure emotional dimension with age and sex-depend increased emotional state, suggesting that underlying distinct sex-dependent neurobiological substrate derangements are also age-dependent, from young to old age in NTg mice but, most importantly, from premorbid to advanced stages of the disease in the 3xTg-AD mice. The results “blind to sex and age” demonstrate the need to analyze both sexes independently. LaFerla’s triple-transgenic mice can provide a model for understanding the mechanistic underpinnings of sex- and AD-disease-dependent modulation of cold (Baeta-Corral et al., 2015) and hot (present work) thermal pain sensitivity and be useful to progress in preclinical studies on pain management in dementia under a gender medicine perspective, with the aim of developing sex-specific analgesic therapies.

## Data Availability Statement

The raw data supporting the conclusions of this article will be made available by the authors, without undue reservation.

## Ethics Statement

The animal study was reviewed and approved by CEEAH and Generalitat de Catalunya.

## Author Contributions

LG-L: conceptualization. TC: performance and analysis of behavior, illustrations, and drafting the manuscript. All authors contributed to the article and approved the submitted version.

## Conflict of Interest

The authors declare that the research was conducted in the absence of any commercial or financial relationships that could be construed as a potential conflict of interest.
